# The Application of circRNA-016901 in Improving the Diagnostic Accuracy of Osteoarthritis

**DOI:** 10.1155/2022/1158562

**Published:** 2022-06-06

**Authors:** Mingchang Du, Shiwen Fan, Ye Liu, Yifan Hao, Jie Guo

**Affiliations:** Orthopedics Department, The Orthopedic Hospital of Shenyang, No. 115 Dongbei Dama Road, Dadong District, Shenyang City, Liaoning Province 110000, China

## Abstract

In clinical practice, osteoarthritis (OA) is frequently misdiagnosed as rheumatoid arthritis (RA) and osteonecrosis (ON), leading to wrong treatment and disease progression. Circular RNA- (circRNA-) 016901 affects the recovery of irradiation-induced injury in the bone, while its role in OA is unclear. This study is aimed at exploring the role of circRNA-016901 in improving the diagnostic accuracy of OA. The present study included patients with OA (*n* = 80), patients with RA (*n* = 80), patients with ON (*n* = 80), and healthy controls (HCs, *n* = 80) to collect plasma samples before and after treatment. RT-qPCR was performed to detect RNA accumulation of circRNA-016901 in plasma samples from all participants. The role of plasma expression of circRNA-016901 in predicting OA was studied with ROC curve analysis. Association between plasma expression of circRNA-016901 and patients' clinical features was analyzed with the chi-squared test. Compared to HCs, increased accumulation of circRNA-016901 was only observed in the OA group, but not in the RA and ON groups before treatment. OA patients were effectively separated from the RA, ON, and HC groups using plasma expression of circRNA-016901 before treatment as a biomarker. Plasma expression of circRNA-016901 was closely associated with OA patients' disease severity. After treatment, decreased plasma expression levels of circRNA-016901 were only observed in OA patients, while no alteration in plasma circRNA-016901 accumulation was observed in the RA and ON groups. In conclusion, circRNA-016901 is accumulated to high levels in OA and may be applied to improve the diagnostic accuracy of OA.

## 1. Introduction

As the most frequently diagnosed arthritis, osteoarthritis (OA) is characterized by joint pain, tenderness, stiffness, and in some cases bone spurs [1, 2]. OA can affect the hands, knees, and hips, leading to the loss of flexibility [3]. OA shows different prevalence in different age and gender groups [4]. Before 45 years old, OA tends to affect more men than women, while most OA patients older than 45 years old are women [4]. Generally, more than 10% of people older than 60 years old are currently suffering from OA and no cure is available [5, 6]. Multiple approaches, such as medication and exercise can be applied to relieve disease symptoms, but the life quality of patients will still be severely affected. Therefore, novel approaches are needed to guide the future management of OA.

Early diagnosis is critical in managing OA since pathological changes of OA are irreversible [7]. However, OA in primary care center is frequently misdiagnosed as rheumatoid arthritis (RA) and osteonecrosis (ON). Therefore, misdiagnosis is common and disease progression is inevitable [8–10]. In effect, about half of the patients with OA can be misdiagnosed as RA during the initial diagnosis [8]. Although some approaches, such as hip measures and implementation of specialized clinics, have been developed to improve the diagnostic accuracy, these approaches are either limited by the low sensitivity/specificity or are affected by different populations [8–10]. Circular RNAs (circRNAs) cannot encode proteins or only encode small peptides, but they can interact with other RNAs or proteins to participate in human diseases, including OA [11–13]. For instance, circRNAs may sponge miRNAs to suppress their activities in human diseases, thereby regulating the expression of target genes [11–13]. circRNAs may also interact with RNA-binding proteins to regulate protein functions [11–13]. Therefore, circRNAs are potential biomarkers for OA. circRNA-016901 has been reported to be involved in irradiation-induced injury in the bone [14], where silencing of circRNA-016901 regulates the miR-1249-5p/HIPK2 axis to attenuate disease progression. However, its role in OA and other bone diseases is unclear. Our preliminary deep sequencing analysis revealed altered expression of circRNA-016901 in OA, but not in RA and ON (Supplemental File 1), suggesting the specific participation of this circRNA in OA. This study explored the role of circRNA-016901 in OA, RA, and ON, with a focus on its application in improving the diagnostic accuracy of OA.

## 2. Materials and Methods

### 2.1. Research Participants

The present research included patients with OA (*n* = 80, 44 females and 36 males, mean age 55.4 ± 7.8 years old), patients with RA (*n* = 80, 44 females and 36 males, mean age 55.6 ± 7.6 years old), patients with ON (*n* = 80, 44 females and 36 males, mean age 55.5 ± 7.7 years old), and healthy controls (HCs, *n* = 80, 44 females and 36 males, mean age 55.6 ± 7.3 years old) at the Orthopedic Hospital of Shenyang after the Ethics Committee of the hospital approved this study. All participants signed the informed consent. These participants were randomly selected from the 522 OA patients, 398 RA patients, 179 ON patients, and 14,992 healthy controls who were admitted at this hospital from May 2020 to May 2021. Patients' inclusion criteria are as follows: (1) newly diagnosed cases, (2) no therapy was initiation prior to admission, and (3) patients were willing to participate. Patients' exclusion criteria are as follows: (1) recurrent cases, (2) patients with blood relationship, and (3) patients complicated with other clinical disorders. OA patients were diagnosed by analyzing X-ray images of the affected joints and symptoms. Diagnostic criteria for OA are as follows: (1) symptoms, such as cool effusions, crepitus, bony enlargement, and decreased range of motion and (2) radiographic hallmarks, such as osteophyte formation, nonuniform joint space loss, cyst formation, and subchondral sclerosis [15]. Patients with RA were diagnosed through a combined approaches including laboratory tests, physical exam, medical history analysis, and imaging tests [16]. ON patients were diagnosed by performing MRI [17]. Participants in the HC group received systemic physiological analysis, and no abnormalities were observed.

### 2.2. Treatment and Plasma Preparation

OA and RA patients were treated with NSAIDs (celecoxib (200 mg per day) or etodolac (600-1,000 mg per day)). RA patients were also treated with prednisone (5 to 10 mg per day). Clarithromycin (250-500 mg per time, twice a day) was used to treat ON patients. Dosages vary across patients. Blood was extracted under a fasting condition before and after the treatment (6 weeks). Blood samples were centrifuged in EDTA tubes at 1,200 *g* for 18 min to collect plasma samples.

### 2.3. RNA Preparation

RNeasy Micro Kit (QIANGEN) was applied to prepare RNA samples from plasma samples. Briefly, cell lysis buffer was mixed with clinical sample to a ratio no less than 10 : 1. Samples were then lysed on ice for at least 30 min and then homogenized through vertex for 10 s. Then, ethanol was added to cell lysate and RNA binding to membrane was performed through centrifugation at 12,000 *g* for 1 min. After washing with buffer for 2 times, empty tubes were further centrifuged at 12,000 *g* for 3 min to completely remove ethanol. Finally, nuclease-free water was added and centrifugation at 12,000 *g* was performed for 2 min to elute RNA.

### 2.4. RNA Analysis and RT-qPCR

Prior to the subsequent analysis, the quality and quantity of RNA samples were analyzed using a 2100 Bioanalyzer. RNA preparation step was repeated on samples with unsatisfied purity (DNA and/or protein contamination) or integrity (RIN < 8.0). Then, RNA samples were subjected to the preparation of cDNA samples using SuperScript™ IV First-Strand Synthesis System (Thermo Fisher). Samples of cDNA were used in qPCR to quantify the relative expression levels of circRNA-016901 with GAPDH as the internal control. Mixture of qPCR was prepared using Maxima SYBR Green/ROX qPCR Master Mix (2X) (Thermo Scientific). The 2-delta delta Ct method was applied for the normalization of Ct values [18]. Primer sequences were as follows: circ-016901 forward, 5′-ACAGCGCTACACTTGTTCCGA-3′ and reverse, 5′-GACGATGCTATCCAGGAGAGGT-3′; GADPH forward, 5′-CACTGAGCAAGAGAGGCCCTAT-3′ and reverse, 5′-GCAGCGAACTTTATTGATGGTATT-3′. All qPCRs were performed on a Bio-Rad CFX96 Real-Time PCR Machine. PCR thermal conditions were as follows: 2 min at 95°C and then 40 cycles of 10 s at 95°C and 50 s at 58°C.

### 2.5. Statistical Methods

Data from two time points and multiple groups were compared by the paired *t*-test and ANOVA Tukey's test to explore differences, respectively. The role of circRNA-016901 in predicting OA was analyzed by performing ROC curve analysis, in which the true positive and negative cases were OA patients and patients with RA or patients with ON or HCs, respectively. Association between plasma expression of circRNA-016901 and patients' clinical features was analyzed with the chi-squared test after the 80 patients were grouped into high- and low-circRNA-016901 level groups (median value as a cutoff value). *P* < 0.05 was statistically significant.

## 3. Results

### 3.1. Differential Expression of circRNA-016901 in Plasma Samples from Four Groups

**Figure 1:**
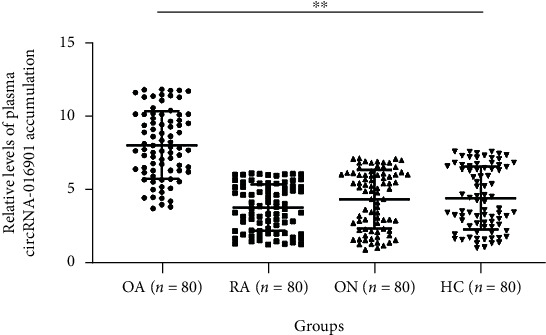
Differential expression of circRNA-016901 in plasma samples from four groups. To analyze the differential accumulation of circRNA-016901 in patients with OA (*n* = 80), patients with RA (*n* = 80), patients with ON (*n* = 80), and healthy controls (HCs, *n* = 80), plasma samples were collected prior to the initiation of therapy. RT-qPCR was applied to determine RNA accumulation levels. ^∗∗^*P* < 0.01.

To analyze the differential expression of circRNA-016901 in patients with OA (*n* = 80), patients with RA (*n* = 80), patients with ON (*n* = 80), and healthy controls (HCs, *n* = 80), plasma samples were collected prior to the initiation of therapy. Expression levels of circRNA-016901 were determined through RT-qPCR, and relative gene expression levels were calculated. Relative expression levels of circRNA-016901 in the OA, RA, ON, and HCs groups were 7.81 ± 3.01, 3.43 ± 1.12, 3.53 ± 1.23, and 3.61 ± 1.32, respectively. RT-qPCR analysis showed increased expression levels of circRNA-016901 in the OA group (1.77-fold) compared to that in HCs, but not in the RA and ON groups before treatment (Figure 1, *P* < 0.05).

### 3.2. The Role of Plasma Expression of circRNA-016901 in Predicting OA

**Figure 2:**
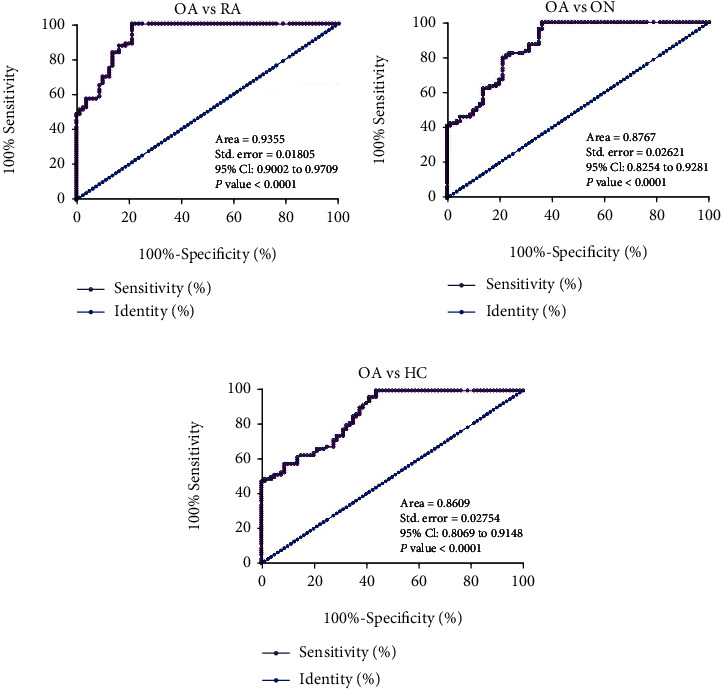
The role of plasma circRNA-016901 in predicting OA. The role of circRNA-016901 in predicting OA was analyzed by performing ROC curve analysis, in which the true positive and negative cases were OA patients and patients with RA (a) or patients with ON (b) or HCs (c), respectively.

The role of circRNA-016901 in predicting OA was analyzed by performing ROC curve analysis, in which the true positive and negative cases were OA patients and patients with RA or patients with ON or HCs, respectively. With plasma expression of circRNA-016901 as a biomarker, OA patients were efficiently separated from RA patients (Figure 2(a)), ON patients (Figure 2(b)), and HCs (Figure 2(c)). Therefore, plasma circRNA-016901 may be applied in clinical practice to improve the diagnostic accuracy of OA.

### 3.3. The Effects of Treatment on the Expression of circRNA-016901 in Plasma Samples from Three Patient Groups

**Figure 3:**
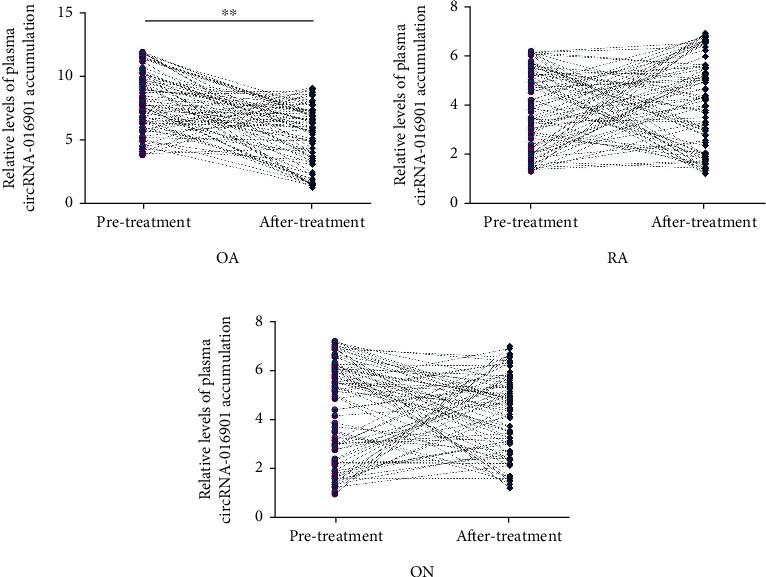
The effects of treatment on the accumulation of circRNA-016901 in plasma samples from three patient groups. Plasma was obtained from the OA, RA, and ON groups at 6 weeks after the initiation of therapy to determine the expression of circRNA-016901. OA, RA, and ON patients were treated by different methods (see details in Materials and Methods). Plasma accumulation of circRNA-016901 was compared before (pretreatment) and after (posttreatment) treatment by performing the paired *t*-test in the OA (a), RA (b), and ON (c) groups. ^∗∗^*P* < 0.01.

Plasma was obtained from all three groups of patients (OA, RA, and ON) at 6 weeks after the initiation of therapy. Plasma accumulation of circRNA-016901 was compared before and after treatment. After treatment, decreased plasma expression levels of circRNA-016901 were only observed in OA patients (Figure 3(a), *P* < 0.01, 1.44-fold), while no significant alteration in plasma expression of circRNA-016901 was observed in the RA (Figure 3(b)) and ON (Figure 3(c)) groups.

### 3.4. Associations between Plasma circRNA-016901 and Patients' Clinical Features

Associations between plasma expression of circRNA-016901 and patients' clinical features were analyzed with the chi-squared test. As shown in Table 1, plasma expression of circRNA-016901 was closely associated with OA patients' disease severity (KL grade), but not gender, age, BMI, and habits of smoking and drinking.

### 3.5. The Role of Plasma Expression of circRNA-016901 in Distinguishing Disease Severity

**Figure 4:**
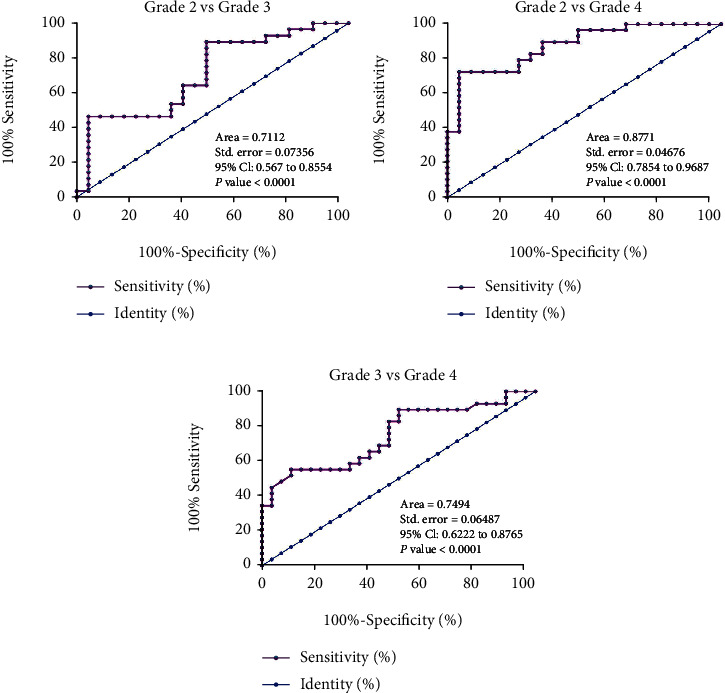
The role of plasma circRNA-016901 in distinguishing disease severity. The 80 patients included 23, 28, and 29 cases at KL grades 2, 3, and 4, respectively. The role of plasma circRNA-016901 in distinguishing grade 2 (true positive cases) from grade 3 (true negative cases, a) and 4 (true negative cases, b) patients and in distinguishing grade 3 (true positive cases) patients from grade 4 (true negative cases, c) patients was studied with ROC curve analysis.

The 80 patients included 23, 28, and 29 cases at KL grades 2, 3, and 4, respectively. The role of plasma expression of circRNA-016901 in distinguishing disease severity was analyzed by performing ROC curve analysis. Using plasma expression of circRNA-016901 as a biomarker, grade 2 (true positive cases) patients were effectively separated from grade 3 (true negative cases, Figure 4(a)) and 4 (true negative cases, Figure 4(b)) patients. Moreover, grade 3 (true positive cases) patients were also effectively separated from grade 4 (true negative cases, Figure 4(c)) patients.

## 4. Discussion

This study investigated the expression of circRNA-016901 in plasma of patients with OA, RA, and ON. Our results demonstrated altered expression of circRNA-016901 in OA plasma samples. Moreover, we also showed that plasma expression of circRNA-016901 may be applied in clinical practice as a biomarker to predict OA, treatment outcomes, and disease severity.

In a recent study, circRNA-016901 is reported to interact with the miR-1249-5p/HIPK2 axis in bone mesenchymal stem cells, and silencing of circRNA-016901 attenuates cell injury induced by irradiation [14]. Since bone mesenchymal stem cells also play critical roles in the development of OA, we speculated that this circRNA may also participate in OA. The present study reported the increased expression levels of circRNA-016901 in OA plasma samples, but not in RA and ON plasma samples compared to that in HC plasma samples. Therefore, circRNA-016901 may specifically participate in OA. However, the role of circRNA-016901 in OA and the mechanisms that mediate its role remain to be further investigated [19]. In addition, the causes of the upregulation of circRNA-016901 in OA are unclear. However, it is known that molecular mechanisms of OA, RA, and ON are quite different, and different genes are involved in disease initiation, development, and progression [20, 21]. Therefore, the different gene expression networks involved in RA may cause the specific upregulation of circRNA-016901.

Although previous studies have reported the application of circRNAs as biomarkers for the diagnosis of OA [22, 23], these studies only included HCs as true negative cases in ROC curve analysis. For instance, using increased serum expression levels of circ_RUNX2 as a biomarker, patients with OA were separated from controls [22]. In another study, the application of blood hsa_circ_0032131 in separating OA patients from the controls has also been investigated [23]. However, OA in primary care center can be misdiagnosed as RA or even ON in many cases. Using only HCs as true negative cases cannot fully explore the clinical value of biomarkers for OA. In this study, we showed that OA patients were effectively separated from RA and ON patients using circRNA-016901 patients. Moreover, decreased plasma expression levels of circRNA-016901 were only observed in OA patients after treatment. In addition, plasma expression of circRNA-016901 can also be used to distinguish OA patients with disease severity. Therefore, detection of plasma expression levels of circRNA-016901 may be applied in clinical practice to improve the diagnosis of OA and its severity. It can also be used as an indicator of disease treatment.

In conclusion, plasma expression of circRNA-016901 was specifically upregulated in OA. Increased plasma expression levels of circRNA-016901 may be applied to improve the diagnosis of OA and used as an indicator of its treatment and disease severity.

## Figures and Tables

**Table 1:** Chi-squared association analysis between plasma circRNA-016901 and patients' clinical features.

Features	Cases	High (*n* = 40)	Low (*n* = 40)	*P*
Gender				0.37
Male	36	20	16	
Female	44	20	24	
Age (years old)				0.65
	38	18	20	
	42	22	20	
BMI (kg/m^2^)				0.65
	34	16	18	
	46	24	22	
Smoking				0.50
Yes	33	18	15	
No	47	22	25	
Drinking				0.37
Yes	42	23	19	
No	38	17	21	
Family history				0.45
Yes	21	12	9	
No	59	28	31	
KL grade				0.004
2	23	6	17	
3	28	13	15	
4	29	21	8	

## Data Availability

The datasets generated during and/or analyzed during the current study are not publicly available but are available from the corresponding authors on reasonable request.

## Supplementary Materials

Supplementary Materials Supplemental File 1: deep sequencing analysis of circRNA-016901 expression in OA, RA, and ON patients and healthy controls (reads per million).
