# Supplementary material for: The Application of circRNA-016901 in Improving the Diagnostic Accuracy of Osteoarthritis
Source: Biomed Res Int. 2022 Jun 6;2022:1158562. doi: 10.1155/2022/1158562 (PMC9192245; doi:10.1155/2022/1158562)
Supplement: Supplementary Materials — Supplemental File 1: deep sequencing analysis of circRNA-016901 expression in OA, RA, and ON patients and healthy controls (reads per million). [file 1158562.f1.docx]

|  | OA (n=10) | RA (n=10) | ON (n=10) | Control (n=10) |
| --- | --- | --- | --- | --- |
|  | 94 | 37 | 45 | 64 |
|  | 115 | 23 | 69 | 70 |
|  | 136 | 80 | 39 | 77 |
|  | 160 | 68 | 76 | 31 |
|  | 169 | 47 | 21 | 20 |
|  | 180 | 45 | 44 | 50 |
|  | 148 | 68 | 79 | 68 |
|  | 143 | 40 | 37 | 84 |
|  | 87 | 41 | 48 | 59 |
|  | 170 | 55 | 52 | 80 |
| Mean±SD | 140.2±32.3 | 50.4±17.3 | 51.0±18.5 | 60.3±21.0 |
